# Possibilities of Using Selected Additive Methods for the Production of Polymer Harmonic Drive Prototypes

**DOI:** 10.3390/ma16114073

**Published:** 2023-05-30

**Authors:** Jacek Pacana, Andrzej Pacana, Rafał Oliwa

**Affiliations:** 1 Faculty of Mechanical Engineering and Aeronautics, Rzeszow University of Technology, al. Powstancow Warszawy 12, 35-959 Rzeszow, Poland; app@prz.edu.pl; 2 Department of Polymer Composites, Rzeszow University of Technology, al. Powstancow Warszawy 6, 35-959 Rzeszow, Poland; oliwa@prz.edu.pl

**Keywords:** 3D printing, polymeric materials, FDM, MEM, harmonic drive, flexspline, mechanical engineering

## Abstract

This article includes an analysis of the possibility of using polymer materials for the production of harmonic drive. The use of additive methods greatly eases and accelerates the manufacturing of the flexspline. In the case of gears made of polymeric materials using rapid prototyping (RP) methods, the problem is often with their mechanical strength. In a harmonic drive, the wheel is uniquely exposed to damage, because during work, it deforms and is additionally loaded with torque. Therefore, numerical calculations were conducted using the finite element method (FEM) in the Abaqus program. As a result, information was obtained on the distribution of stresses in the flexspline and their maximum values. On this basis, it was possible to determine whether a flexspline made of specific polymers could be used in commercial harmonic drives or whether they were only adequate for the production of prototypes.

## 1. Introduction

Improving products as well as production processes is necessary to ensure the growing expectations of customers. Development research is carried out permanently in many companies and practically applies to every product. One of such directions of improvement is the possibility of using polymeric materials for the production of a harmonic drive [1,2,3].

Currently available are harmonic drives manufactured completely from polymers [4,5] or in which only some components are made with polymers [6,7]. The components of these gearboxes are manufactured industrially by mold injection. Such technology is expensive in implementation and makes it difficult to introduce changes and modifications to the structure. However, harmonic drives are much cheaper than those of steel. Therefore, they are used successfully where they do not transmit large torques, such as in optics, measuring, robotics, and control devices [1,8,9]. Both the major world manufacturers and scientific centers conduct research aimed at improving the design of harmonic drives [5,7]. New design solutions must be tested, preferably with prototypes, before deployment. For laboratory research, harmonics drives can be made quickly and cheaply from polymers using 3D-printing methods [10,11]. 

**Figure 1:**
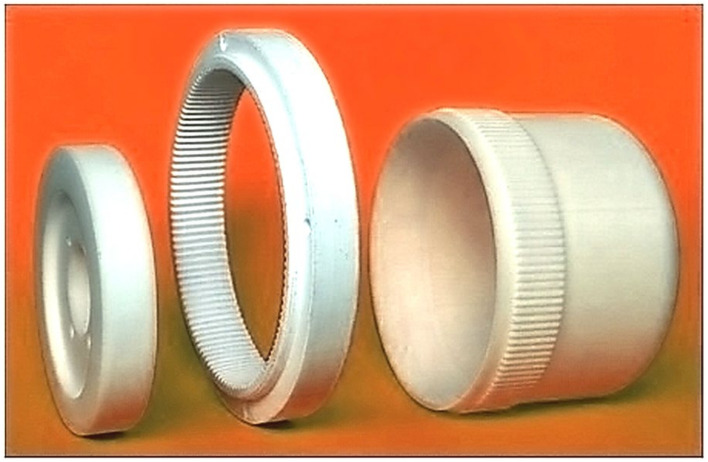
Models of the main components of the harmonic drive made by the FDM method.

The harmonic drive has a very complicated design and a principle of work [3,10]. Its main components are the flexspline, the circular spline, and the wave generator, which are shown in Figure 1. In a harmonic drive, the wave generator deforms the flexspline to a form that ensures correct meshing with the circular spline. A rotating generator causes the waves to move around the circumference of the flexspline and transfer the torque to the circular spline. In gears of this type, many pairs of teeth of both wheels remain in the mesh at the same time, affecting their high load capacity and kinematic accuracy. The advantages of these gears are also the possibility of obtaining a large ratio with small dimensions and quietness.

The flexspline is the most loaded element of the harmonic drive, and at the same time, it is also the most complicated construct. The elongated shape of the flexspline body sleeve causes the need for additional specialized tooling and tools in its manufacture. Harmonic drives are most often made of steel, but in many cases, such high strength is not required from them. Often, wave gears are used in precision mechanisms, measuring devices, or to control and regulate valves, where their high load capacity is not required. More important in such cases is the high kinematic accuracy of the gear ratio, low backlash, or low weight of the structure [11,12]. The use of additive methods and polymers would definitely improve the production of flexspline and reduce costs. Incremental methods allow us to make elements from various materials with different strength properties.

Sometimes, even within one method, it is possible to use different materials. However, for the production of prototype, polymers are mainly used, but metals, technical ceramics, wood, rubber, or composites on a metallic or polymer matrix are also used [13,14,15,16]. The continuous development of polymer materials enriched with nanocomposites allows influencing the strength properties of the manufactured models to a very large extent. Appropriate modification of the parameters of the basing material may also allow the use of models produced by additive methods in contaminated or corrosive environments, in vacuum, or at high or low temperatures.

Rapid prototyping methods have different techniques for building a model, which mainly affects the strength and durability of the manufactured elements. The outer surface quality of the prototypes also depends on the technology used [17,18] and is often not satisfactory.

Often, the availability of the rapid prototyping method is decisive when choosing a technology for manufacturing machine components. However, it should be included that a given production method forces the use of a specific material. This affects the quality of the internal structure and exterior surface of the models. It is also necessary to pay attention to the different strength or temperature parameters of materials used in applied methods of rapid prototyping.

In most cases, the models obtained by RP methods allow for their subsequent machining. Thanks to this, it is possible to obtain finished products with the required dimensional accuracy. A big advantage of using additive methods is also the possibility of making a flexspline with a sleeve in a shape other than standard cylindrical. Modifications to the geometry of the thin-walled body of the flexspline can increase its strength and thus the durability of the entire harmonic drive [19].

The design of new construction solutions should begin with accurate analytical calculations. On their basis, the needed shape of the flexspline is modeled in programs using computer-aided design methods (CAD). The next step of design is most often based on numerical calculations performed using the finite element method (FEM). This calculation method allows you to take into account many different models without the need to make their prototypes and carry out costly bench tests. Using numerical models, it is possible to assess the impact of different materials or operating conditions much faster as well as to analyze the legitimacy of using different design solutions for the tested part. Because the flexspline is an element with a complicated structure and working conditions, it is often necessary to use simplified and limited models [20]. Therefore, an analysis of the strength of wave gears made of materials used in popular rapid prototyping methods was carried out.

## 2. Applied Methods and Tools

Among rapid prototyping techniques, new methods are still emerging, and existing ones are constantly being developed. Changes are oriented at accelerating the production process, improving its quality and reducing costs. This provides the possibility of using rapid prototyping methods in the future also in series production. There are many incremental methods, but only those that the authors have the opportunity to use for manufacturing functional prototypes of the harmonic drive were selected for the realized analysis. Verification of numerical calculations with the use of physical models will be the subject of future bench tests. For the calculation, models were included, which are components of the harmonic drive produced by the following methods:

- Selective Laser Sintering (SLS);
- Fused Deposition Modeling (FDM);
- Jetting System (JS);
- Stereolithography (SLA).

Using the SLS method, the physical model is performed by a high-power laser that melts the thermoplastic material, combining it with the previous layers [21]. First, a layer of thermoplastic material is distributed on the working platform, and the laser beam moving along a specific trajectory melts the powdered medium. When curing the outline of the current layer, the laser also melts the previous one, causing them to combine. The machine then lowers the ladle with the working material by the thickness of the layer and applies another, and the whole process is repeated from the beginning. In the manufacturing process, the model is constantly supported by the surrounding powder, so there is no need for additional supports. 

The advantages of this method are also the high durability of models, high quality of external surfaces, and the possibility of making “ready-made” models with very complex shapes. The disadvantage of this method is the need for long-term cooling of the models before removing them from the working space. This method was chosen for the analysis because the manufactured prototypes produced with it can achieve the material density of the finished products obtained by industrial methods. 

The FDM method consists of creating a model from a material supplied in a semi-liquid state. The filament (i.e., the material in the form of threads) is pumped through a nozzle that has the ability to move precisely in the horizontal plane. The modeling of subsequent layers of the physical model is realized by vertical movement of the working platform [22]. Since the applied material is melted, it combines with the previous layers of material to form a uniform structure. In order to achieve high product quality, it is very important to properly control the temperature of the nozzle and thus also the material to be joined. In the FDM method, two nozzles can be used separately for the building of the supports and model. If only one nozzle is used both to create the proper model and to build supports, it is called MEM [23].

Many different materials can be used, but models obtained with this method from ABS, especially modified ABS, have high dimensional accuracy. The durability of models obtained by the FDM method is high, with parameters similar to those of products obtained by traditional methods [24]. 

The Jetting System (JS) involves a layered application of photosetting materials from the print head. The resin beam that flows out of the print head is cured with light. JS printing involves building a proper model and making supports that are removed at the end of the process [25]. The model obtained in the CAD program is reproduced in layers. The print head together with the UV lamp moves horizontally until the curing of the layer is completed. Then, the table is lowered, together with the manufactured element, by the thickness of the layer, and the process of feeding and fixing the resin begins again. 

The JS method uses polymer photocuring resins, thanks to which prototypes with significantly different properties can be obtained. Depending on your expectations, you can choose a material with high strength, rigidity, or flexibility, resistant to moisture, or allowing models to be obtained with high dimensional accuracy. Poly-Jet technology is used to produce prototypes that are used, m.in, in the automotive, aerospace, and medical industries [26]. 

In the SLA method, the physical model is built by a laser beam irradiating the surface of the resin in the place where it is to be cured. In the next stages, the contours of the layer are hardened, the interior of the cross-section is stiffened, the working platform is lowered, the next layer of uncured resin is applied and leveled, and the next layer is cured again.

In this method, it is necessary to make supports in the shape of thin stamens, which must be removed after the completion of the modeling process. Models are made of photocurable polymer resins, which allow obtaining models with the highest accuracy, but it depends largely on the proper calibration of the device and the appropriate preparation and positioning of the model relative to the direction of growth of the model [27,28].

The described methods allow us to obtain homogeneous products with strength characteristics such as for the base material. Table 1 shows the selected properties of the polymers used in the numerical calculations. Polyamide PA 2200 was chosen as the material used in the SLS method, while ABS was chosen for the FDM method. The resins used in subsequent methods are FullCure 720 for the JS method and SL5220 for stereolithography (SLA).

## 3. Calculations

Thanks to numerical calculations realized using the FEM method, it is possible to perform strength calculations for gear components made of different materials. In the Abaqus program, it was possible to simulate real working conditions, perform calculations, and finally directly compare the results for all the analyzed materials.

In order for the analysis of the results to be reliable, exactly the same calculation models were assumed for all calculation variants, and identical boundary conditions were defined. The only difference was in the physical properties, which were individually determined in each case for the material according to the parameters given in Table 1. Since an important aspect of the calculation was the deformation of the flexible under load, it was decided to use a full harmonic drive model.

Calculation models of harmonic drive were subjected to a discretization process mainly by means of hexahedral square elements (hexagonal 20 node quadratic brick—C3D20R). For the flexspline model 375,427 elements were defined, for the circular spline, 285,554 elements were defined, and for the wave generator, 97,254 elements were defined. For both gears, the toothed wheel rim forced a higher density of finite element mesh. In these areas, between cooperating models, also a contact of type surface to surface and friction conditions fine sliding was defined. A second pair of contact surfaces was defined between the inner diameter of the flexspline and the generator. This corresponds to the actual conditions of cooperation of these elements. In the calculations, it was assumed that the deformations of the elements are small, i.e., they remain throughout the entire operation in the linear elastic deformation region [33,34], which has also been previously stated by means of conducted studies [14,17]. In addition, ambient conditions related to temperature or vibrations do not change the assumed strength characteristics of the tested materials, and thus there are no plastic deformations [35]. The deformations of the elements analyzed are small, while the displacements of some of them are large, which was taken into account for the analysis carried out in the Abaqus program [36]. 

However, it was necessary to perform some preparatory operations, without which it would not have been possible to execute the correct calculations.

**Figure 2:**
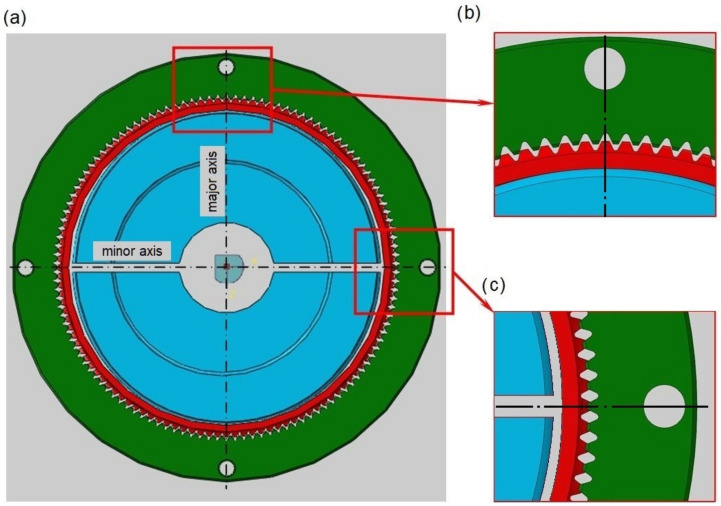
Model of the harmonic drive prepared for FEM calculations: (**a**) general view, (**b**) enlargement of the meshing area in a major axis, (**c**) enlargement of the model region in a minor axis.

The strength analysis of the wave gear was carried out in two steps: initial and main. The first step of the calculation consisted of deforming the flexspline by the generator until its correct meshing with the circular spline in their major axis. It was decided to use a cam generator, because it provides control of the deformation form of the flexspline around the whole circumference. The generator was divided along its minor axis, which is visible on the calculation model shown in Figure 2.

In the first step of the calculation, the halves of the radial generator model were moved along its major axis. By defining the contact surfaces between the generator and the flexspline, the required deformation form of the flexspline and the correct meshing of both gears were obtained. In the second step, the harmonic drive was loaded with a torque of 10 Nm, which was applied to the circular spline. Such a value was adopted on the basis of bench tests realized by the authors, because it allowed many hours of work of prototypes without obvious damage. Among the harmonic drives offered for sale, there are similar ones for which the nominal load is 4 Nm and the maximum is 20 Nm [5]. On the models of both gears, contact surfaces were defined on the tooth flanks. It was also assumed that during the operation of the harmonic drive, the fixed element is the flexspline, and the mounting is defined at its bottom.

In addition to the discussed problems, the preparation of the computational model was no longer difficult and was performed in the following steps: importing models, preparing the assembly, defining boundary conditions and load, determining contact surfaces, generating a finite element mesh, and preparing a file for solver. The harmonic drive model was very complex, so the realization of individual tasks was made with special attention. The calculations were made using the same model for all four polymer materials.

However, it should be noted that despite its many advantages, the finite element method used in the analysis is a simplified calculation based on discrete models. It is not possible to take into account very complex operating conditions, contact, the influence of certain environmental conditions or material parameters, as well as random material heterogeneities. In addition, for the analyzed cases, some simplifications were adopted, for example, their material structure and density being homogeneous throughout the models. However, the key problem of the analysis was the comparison of the four material solutions presented. Since the same calculation conditions were maintained in all cases, the effect of the method on errors in the obtained results was negligible. Despite its obvious limitations, the finite element method (FEM) is a recognized and successfully used numerical computational method used in engineering design.

## 4. Results

**Figure 3:**
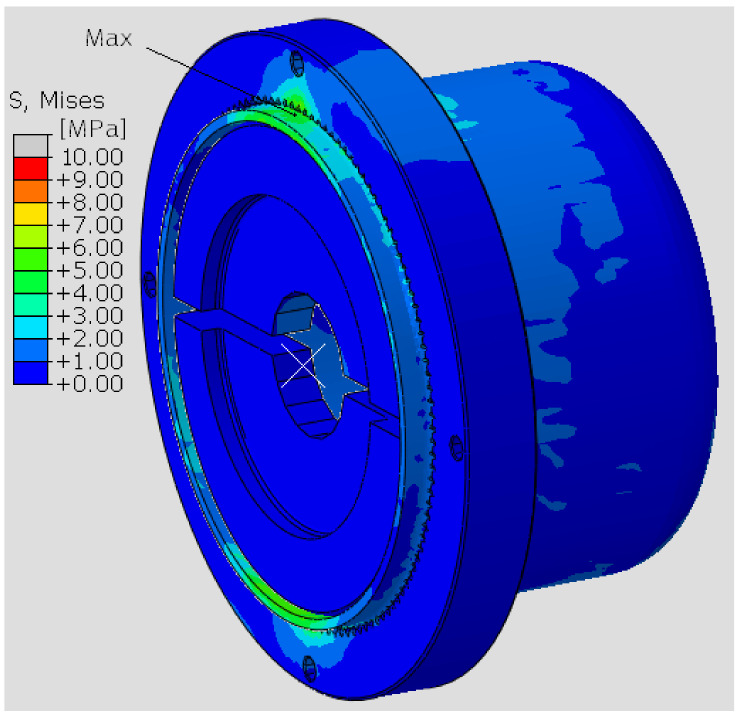
The stress distribution on the flexspline model.

Numerical calculations carried out in the Abaqus program allowed obtaining an extensive range of results. The results on the distribution of the reduced stresses are shown in Figure 3.

The graphical presentation of the results confirms that the most loaded element of the harmonic drive is the flexspline. Clearly, on the surface of this model, there are visible areas of deformation on the initially cylindrical body sleeve. At the same time, it can be observed that the generator model has a single blue color, which means that the whole moves as one undeformed solid.

**Figure 4:**
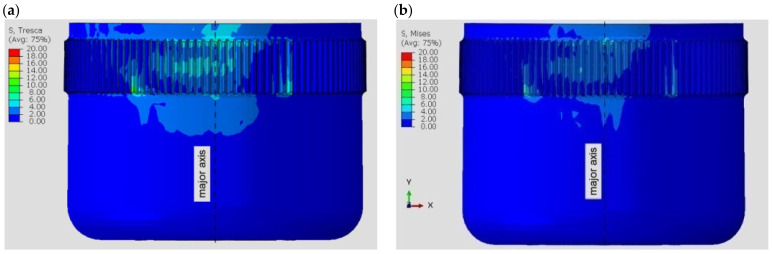
Stress distribution in the polymer flexspline model: (**a**) ABS—acrylonitrile–butadiene–styrene, (**b**) PA2200—polyamide.

For various materials, different stress distributions were obtained in the flexspline model. The discrepancies are due to the properties of the material defined for the calculations. Figure 4 shows a view of the flexspline from the side of the major axis of the generator for the two polymers included in the calculations. Differences in the stress distribution are clearly visible, and there are higher stress values with ABS.

**Figure 5:**
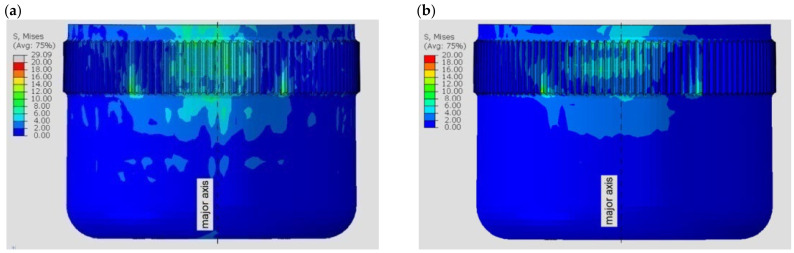
Stress distribution in the resin flexspline model: (**a**) FullCure 720, (**b**) SL5220.

Figure 5 shows analogous results obtained for the next two materials. In the case of resins, higher values were obtained for the model made in the Poly-Jet method, i.e., FullCure 720.

For all computational models, the highest stresses are visible in the area near the major axis of the generator. This is due to the compound stresses state of both deformation, i.e., bending of the flexspline, and contact on the teeth flange by load. It can also be observed that the stress distribution is not symmetric relative to the axis of the model. In Figure 4 and Figure 5, a slight displacement of the area of the higher values to the left of the major axis of the flexspline is visible. The relocation is in the opposite direction to the defined torque direction. The displacement of the area of cooperation of the harmonic drive, relative to its main axes, is known from the literature [2,3,37,38], and numerical calculations have confirmed this feature. However, the displacement of this area is not significant, because the stresses that occur in the flexspline are mainly the result of its deformation.

**Figure 6:**
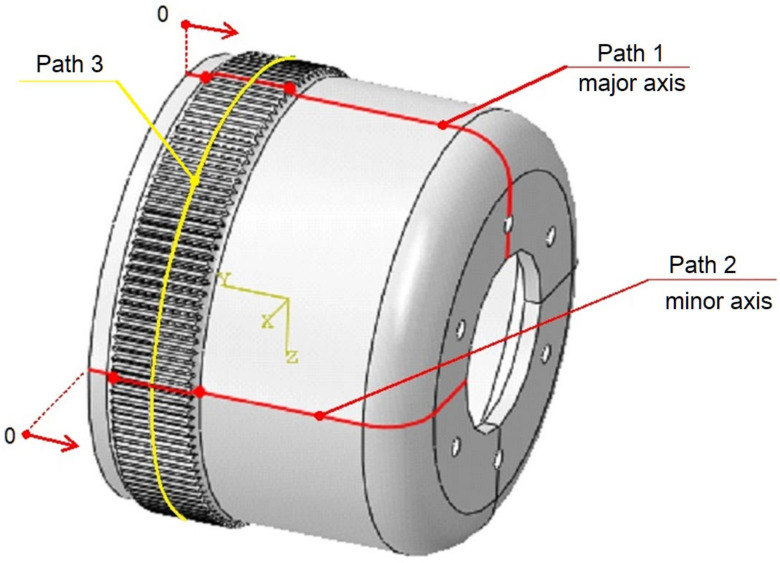
Measurement paths, defined for flexspline in the Abaqus postprocessor.

To more accurately compare the stresses present in the model, their values read along the paths defined on the flexspline model were compared. To do this, the Path function in the Abaqus Visualization module was used. The paths were defined by all nodes of the finite element mesh, lying in the major and minor axes of the generator, as shown in Figure 6. This figure also presents the location of path 3, the use of which is described later in this article. 

The beginning of the path marked as “0” was assumed to be at the edge of the flexspline, from the side of the toothed wheel rim, and the location of the next nodes was determined as the distance from this point.

**Figure 7:**
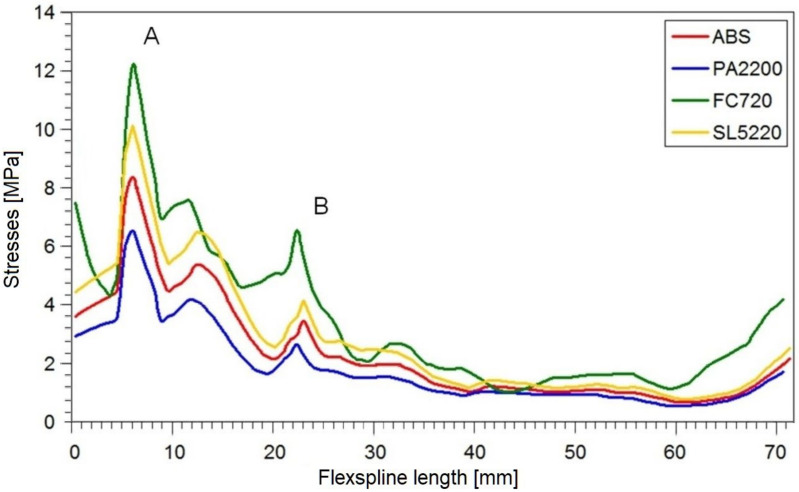
Stress distribution on a major axis for a loaded flexspline.

**Figure 8:**
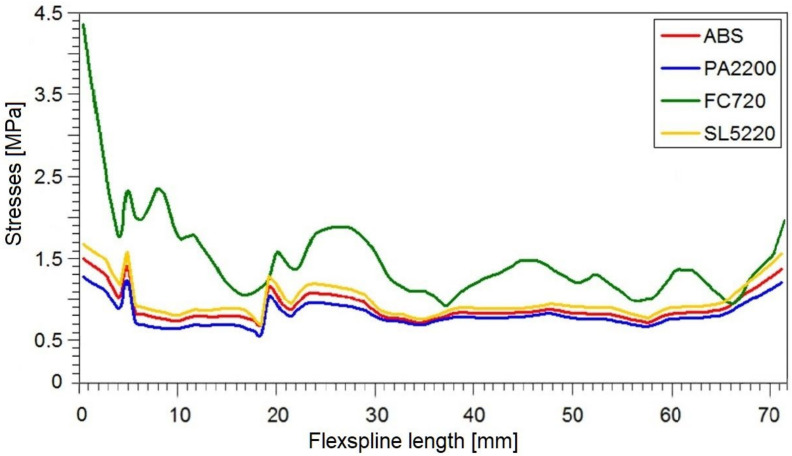
Stress distribution on a minor axis for a loaded flexspline.

In this way, the values on the abscissa axis (x) were also determined in the stress diagrams created shown in Figure 7 and Figure 8. It is clear that for all variants of the material analyzed, the stress distribution on the flexspline has a similar form. The highest values are found at the edge of the toothed wheel rim, located at the end of the flexible sleeve, which is marked in Figure 6 as “A”. This shape of the graph is strongly influenced by surface pressures resulting from the meshing of the teeth of both gear wheels. Another area of higher values was located in the place marked with “B”. In this area, the local increase in stress is caused by a change in the thickness of the flexspline between the toothed wheel rim and the thinner sleeve body. For all the analyzed materials, the highest values occurred in the case of FullCure720 slightly lower for the second resin. The stresses for ABS were about 30% lower, and the lowest were read for polyamide, and they accounted for about half the magnitude obtained for resin.

The reduced stresses read on path 2 along the minor axis had a slightly different course than in the large wheel axis. The graphs for all models were irregularly shaped, and no characteristic fragments could be observed. The only thing worth noting is the fact that by far, the highest stress values occurred at the very beginning of the graph, i.e., at the outer edge of the susceptible sleeve. In diagram 8 on the minor axis, for all tested materials, significantly lower stress values were obtained than in the case of a major axis.

The stresses in the body of the flexspline are therefore directly related to its deformation. In the major axis of the generator, where the radial displacements are greatest, the maximum stress values occur. The second area with large radial displacement values is the minor axis of the flexspline. In this case, the stress values are slightly lower, since the radial displacements in the real flexspline are smaller than in the major axis. The analytically determined displacement values should be the same in both characteristic axes, but there are differences in the real model. In addition, in numerical calculations, other stress values are visible, which is the result of the missing of support of the flexspline by the wave generator in the minor axis. 

Determining the stress distribution on the body of the flexspline is very important in designing the shape of the flexspline sleeve. This is especially true because the values and distribution of these stresses affect the stresses that occur on the rim of the toothed wheel rim. However, stresses on the toothed wheel rim itself are definitely crucial for the strength of the flexspline. From Figure 7 and Figure 8, it can be deduced that this is the most loaded fragment of the flexspline. The available results of the bench tests confirm that flexspline damage most often appears in this area [39].

**Figure 9:**
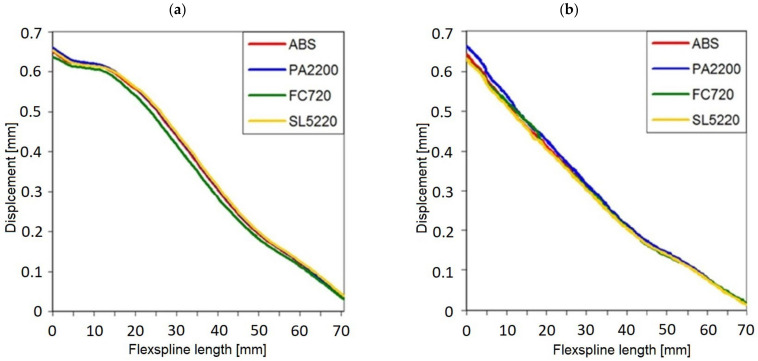
Deformations for a loaded flexspline: (**a**) on a major axis, (**b**) on a minor axis.

The numerical calculations of FEM also allowed determining the deformation form of flexspline models made of various materials. Because stresses are dependent on displacements, the highest stress values can be expected in the areas where the greatest deformation has occurred. Figure 9 presents the absolute values of the displacements of the nodes of the finite element grid on the major and minor axes of the wave generator.

The results are presented along the same paths 1 and 2 shown in Figure 6. The graphs were created according to the same principles as for Figure 7 and Figure 8, and the results were read in the same nodes located in the previously described cross-sections.

In all analyzed calculation cases corresponding to subsequent materials, the plot runs are almost the same. Thus, the radial deformation of the body of the flexspline does not depend on the material used but only results from the impact of the wave generator. In the case of a minor axis, the displacement plots have an almost linear course, while in the case of a major axis, the initial segment of the graph is more horizontal. This is due to the direct impact of the generator on the inner diameter of the flexspline, which at the same time is blocked from the outside by the toothed rim of the circular spline. In the small axis, there is only deformation by the generator, while the teeth of the flexspline and circular spline pass with the clearance, so they do not cause additional deformation.

The task to investigate was also the effect of the torque load on the deformations in key areas of the harmonic drive. Therefore, on the same paths as for the graphs in Figure 9, the values for the flexspline without load were read. It was observed that the graphs for the flexspline only deformed by a wave generator compared to the same model additionally loaded with torque differ slightly. The largest differences in the displacement of the flexspline body in its major axis after load were noted for FullCure 720 resin. They were most prominent in the middle of the length of the flexspline sleeve body, and they amounted to 0.02 mm. The least susceptible to additional torque load was the flexspline model made of resin SL5220, for which the maximum differences in displacement values for specific nodes in the major axis did not differ by more than 1.25%. This situation is the result of close cooperation of the toothed rim of the flexspline with the circular spline and simultaneous support by the wave generator.

In the minor axis (Figure 9b), there is also a change in deformation due to the load on the gear but of a slightly different nature. Due to the missing of support, the greatest differences in deformation occur on the edge of the flexspline from the side of the toothed rim. However, they do not have a direct impact on the kinematic accuracy of the harmonic drive, because the teeth in the minor axis are not involved in the torque transmission.

The values of secondary deformations are not without influence on the overall kinematic accuracy of harmonic drive. When designing a harmonic drive for precision applications, it is important to choose a material that is not sensitive to deformation of the thin-walled flexspline. Therefore, the FullCure720 resin used in the JS method should be eliminated from the analyzed materials.

During operation, the flexspline is exposed to multiplane deformation, but for correct cooperation of the teeth, the correct form of deformation of the toothed rim is crucial. In order to precisely determine it, it is necessary to determine the displacement of specific points located in the neutral layer of the flexspline under the toothed rim.

Traditionally, stresses in the area of the toothed wheel rim are calculated analytically by the formulas based on simplified geometry [3,39,40]. Approximately, the toothed wheel rim can then be specified as a ring deformed by a cam generator. This ring is influenced by the bending moment Mg(φ) depending on the angle φ measured from the major axis of the generator. The radial deformations of the ring are small in relation to its diameter, so the stresses do not exceed the limit of applicability of Hooke’s law. Radial displacement values (w) of any point around the circumference of the flexspline can be determined from the relationship [40]

(1) $$\frac{d^{2}w}{d\varphi^{2}}+w=-\frac{M_{g}\left(\varphi \right)R^{2}}{EI_{x}}$$

where:

*w* radial displacement of the neutral layer of the flexspline;

*φ* angle measured from the major axis of the generator;

*R* radius of the inert layer of the flexspline;

*E* Young’s modulus;

*I_x_* moment of inertia, the cross-section of the wheel body under the toothed wheel rim, which is

(2) $$I_{x}=\frac{h_{1}^{3}b_{w}}{12}$$

where

*b_w_* width of the gear rim;

*h_1_* thickness of the body under the toothed wheel rim.

However, in order to accurately calculate the stresses in the flexspline with a toothed rim, it is not enough to use this simplified method. When determining stresses, it is necessary to take into account their interaction in the teeth up to a some height greater than the thickness of the body under the toothing wheel rim. As a result of the torque (T) load on the teeth located in the meshing area, variable forces cause them to bend. The area of the tooth meshing is limited by an angle φi = (18° ÷ 26°), which is placed asymmetrically relative to the major axis of the generator [2,39,40]. Such complicated calculations are rarely resolved analytically, but the finite element method proves perfectly in this work. In the case of the analyzed harmonic drive, we also used numerical calculations with the FEM method to determine the distribution and stress level in the toothed wheel rim.

**Figure 10:**
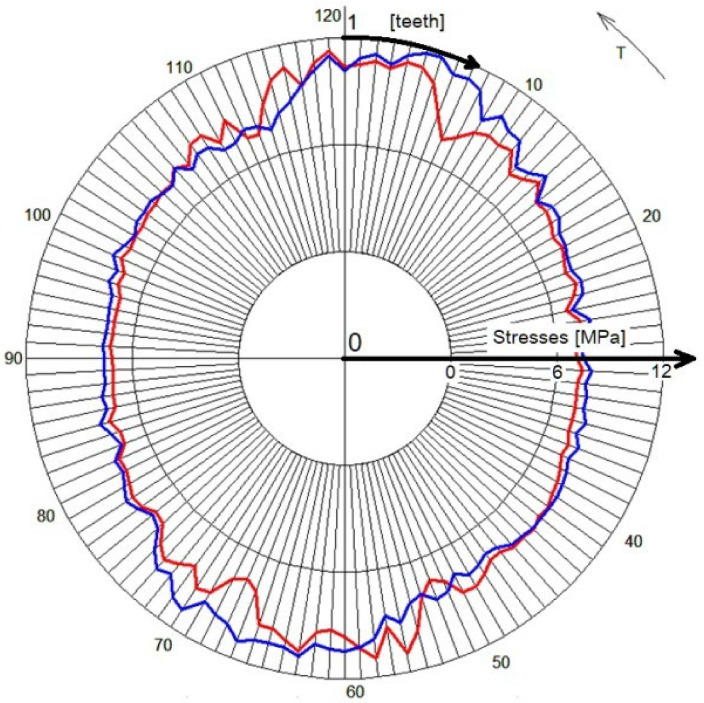
Stresses on the toothed rim of the flexspline made of FullCure 720.

The stress distribution on the flexspline models, presented in Figure 4 and Figure 5, allows locating areas of higher stresses, but only the exact values read in the key areas of the model allow determining its strength. One of the places that determines the strength of a flexspline is the tooth spaces. There is a concentration of stresses in them, and often in the tooth space there is a crack causing the destruction of the flexspline and damage of the harmonic drive [41,42]. This key area of all the models analyzed, differing only in material, was also studied. To accurately locate dangerous areas, stress diagrams were created for subsequent tooth spaces around the circumferent of the flexspline. Stress values were read at the bottoms of subsequent tooth spaces on a cross-section passing through the middle of the width of the toothed wheel rim of the flexspline. The position of the section is shown in Figure 6 as path 3. Since a very similar graph shape was obtained for all four analyzed materials, it was decided to show one of them regarding FullCure 720. Figure 10 shows a radar graph of the stresses read in all 120 spaces for the flexspline model.

In Figure 10, red is used for the toothed wheel rim deformed by the generator, while blue is used for the stresses after the model is loaded by torque. For a flexspline only deformed by a wave generator, the graph is symmetric about the main axes of them. The torque added to the harmonic drive causes the area of higher stresses to shift in the direction opposite to the operation of the load. According to recommendations from the literature [11,22,23] in analytical calculations for the next teeth located directly in the meshing area, the forces values are determined proportionally. Stresses are not evenly distributed, so the approximate determination of the value of the forces that load the teeth does not correspond to the real working conditions. It is therefore necessary to use advanced calculation methods such as FEM which allow one to determine the deformation of the flexspline to be very precise.

The use of numerical methods is also advisable because they use three-dimensional models that allow a precise simulation of working together components of harmonic drive. The high quality of the results is due to the fact that for the flexspline, the forces operating on the teeth are not determined individually; only the load is introduced through their real meshing.

**Figure 11:**
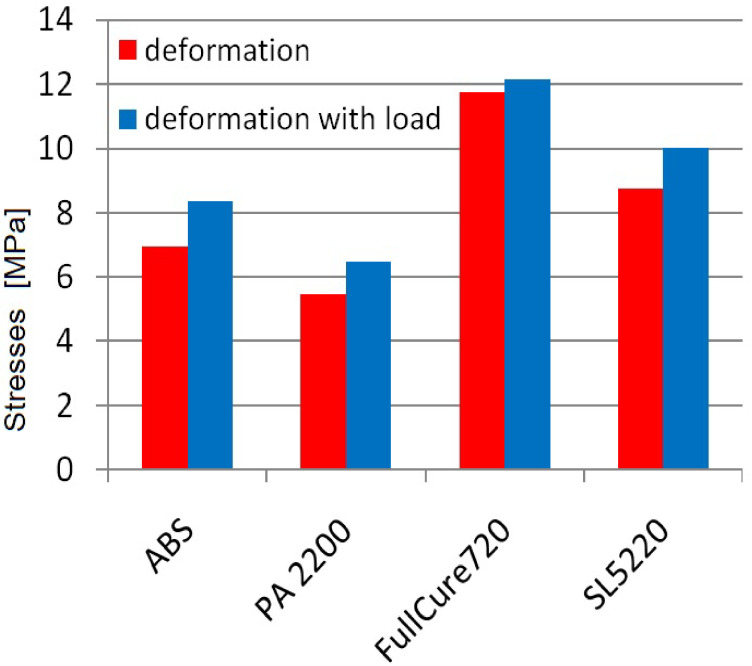
Maximum stresses on the flexspline model.

Figure 11 shows a graph of the maximum stresses read for all four calculation models on path 3. This is accentuated by the situation in which the flexspline was only deformed by the generator and after its loading. Clearly, the highest values occurred with FullCure 720 resin in both load cases. For the other two materials, intermediate values were obtained from which the SL5220 had stress outputs that were about 25% higher than for ABS in both cases of loads. 

The stresses did not reach a level that could damage the components on any of the test materials. By comparing the calculated maximum stresses with the tensile strength given in Table 1, it is possible to check which strength reserve was obtained for each of the materials. For the polymer PA2200, the lowest effort level was obtained, and in this case, the stresses were the lowest. The next wasSL5220 resin and ABS, and the most intense effort has proved the element made of FullCure720 resin. This corresponds to the gradation of the maximum values read on the basis of FEM calculations and depends mainly on the properties of the tested materials.

## 5. Applications

The search for innovative construction and technological solutions is a dominant feature of developing enterprises. This development also applies to harmonic drives [41,43]. Because of their advantages, they are most often used in control and adjustment mechanisms, but they are also used in manipulators and machine tools. Their high kinematic accuracy and the possibility of obtaining large gear ratios on one stage distinguish them from other gear transmissions. Thanks to these advantages, classic steel wave gears have been used in space vehicles. Unfortunately, the design of a harmonic drive is also complicated and causes the production of steel flexspline to classical methods to be a big problem. If the harmonic drives work without a heavy load, it would be recommended to make their elements from polymeric materials. Their strength is lower than in the case of the use of steel flexsplines, but the benefits of easier manufacturing technology are very large. Polymer flexsplines can be made using incremental methods, which reduces costs and expands the range of possible use of harmonic drives. Selected incremental methods enable a partially automated production of elements also in an environment inaccessible to humans. Therefore, it is necessary to study the possibility of producing functional elements of machines in space using polymer materials using rapid prototyping methods. Additive methods can be used only with some materials, which is why this article presents an analysis of the possibility of using selected methods of rapid prototyping and chosen materials for the production of components of harmonic drive. The results of numerical calculations in the form of stress distribution on the surface of flexspline models were analyzed. The focus was mainly on the flexspline, because it is this element of the harmonic drive that is most loaded and most often damaged during operation. Analytical calculations of the harmonic drive geometry of the standard construction were realized, and its solid models were prepared in the CAD program. Next, their construction was simplified and adapted to numerical calculations, which were made based on the finite element method in the Abaqus program. The analysis considered four materials that are commonly used in rapid prototyping methods. Two resins FullCure 720s for JS and SL5220 stereolithography (SLA) are included. Two more materials are polyamide PA 2200 for the SLS laser powder sintering method and acrylonitrile–butadiene–styrene (ABS) for 3D printing (FDM). Numerical calculations for all materials used the same model, and the differences only concern the strength properties.

In order to precisely determine the effect of the load on the distribution and stress values in the toothed wheel rim, on the basis of numerical calculations, the graphs were created. They compared the stress values for a flexspline only deformed by a wave generator and after its load with a torque. For presentation, a radar-type graph was selected so that you can clearly see the asymmetry of stresses resulting from the action of load. The characteristic displacement of the maximum values in the opposite direction to the working moment, as in the example graph (Figure 10), was the same for all calculation models. However, as can be seen from Figure 11, the torque action only slightly increased the maximum stress values. Accurate calculations of such complex multi-directionally deformed models with a load that additionally affects their deformation is possible only with the use of computer computational methods such as FEM.

## 6. Conclusions

The aim of the study was to determine the suitability of selected polymeric materials for the production of harmonic drives. The main assumption of the analysis was the possibility of making transmission elements using rapid prototyping methods. The use of incremental methods significantly accelerates and facilitates the production of prototypes for the needs of bench tests. An important developmental argument for the use of additive methods is to examine their suitability for the manufacture of machine components and thus harmonic drives in space or adverse environments. Determining the strength of prototypes of wave gears made of different polymers gives the answer of whether they are suitable for the production of finished products. 

The results obtained in the calculations show that harmonic drives made of some polymeric materials can find practical applications. Due to the obtained results, but also the conditions of the manufacturing method, the most promising results were obtained for harmonic drives made of ABS I PA2200. The use of resins results in higher stress values in the flexspline and is impossible to use in space, because they are in liquid form before hardening. 

The analysis assumes that harmonic drives made of polymeric materials will be used in the control and regulation mechanism of the mirror or antenna, where the load is small. A higher torque value would cause an obvious increase in the stress value, and too much could even cause the destruction of the harmonic drive. However, in the measuring mechanisms of control or setting devices there are no large loads, and harmonic drives with elements made of polymer can be successfully used. 

However, the final conclusions regarding the applicability of the analyzed methods and materials should also be supported by bench tests using physical models. Experiments allow you to verify the previously obtained results of numerical calculations and check the functionality of models made with rapid prototyping methods. Examples of such studies of harmonic drive prototypes can be found in earlier works of the authors [14,17,44,45]. 

An additional advantage of this solution is the fact that a new harmonic drive or its element can be produced quickly and cheaply using rapid prototyping methods. The shape of its components, especially flexspline, is very complicated, and it is difficult to perform by traditional machining methods. The use of additive methods allows the production of components of harmonic drives with complicated shapes. If necessary, the shape of the flexspline can also be quickly modified, and another functional model can be produced. Further research may concern the possibility of using additive methods to join polymer plastics with other materials, e.g., steel or carbon fibers, in order to increase the strength of models. The realized analysis may also be an important introduction in the study of the possibility of using polymer composites for the production of harmonic drives using rapid prototyping methods.

## Figures and Tables

**Table 1:** Selected physical parameters of materials included in FEM analysis [29,30,31,32].

Parameter		Material			
Name	Unit	PA 2200	ABS	FullCure720	SL5220
		Mechanical properties			
Young’s modulus (E)	[MPa]	1700	2200	2870	2700
Tensile strength (Rm)	[MPa]	48	44	60.3	62
Strength at break (ε),	%	20	9	20	8,3
Flexural modulus (G),	[MPa]	1500	2100	1718	2950
Shore D hardness (15s)	-	75	78	83	86
		Thermal properties			
Melting temperature (20 °C/min)	°C	176	200	61	53
Vicat softening temperature (50 °C/h 50N)	°C	163	108	48.4	42

## Data Availability

Not applicable.

## References

[1] Senthil T.S., Ohmsakthi vel R., Puviyarasan M., Babu S.R., Surakasi R., Sampath B., Keshavamurthy R., Tambrallimath V., Davim J. (2023). Industrial Robot-Integrated Fused Deposition Modelling for the 3D Printing Process. Development, Properties, and Industrial Applications of 3D Printed Polymer Composites.

[2] Dudley D.W. (1962). Harmonic Drive Arrangements. Gear Handbook.

[3] Shen Y., Ye Q. (1985). Theory and Design of Harmonic Drive Gearing.

[4] Harmonic Drive SE https://harmonicdrive.de/fileadmin/user_upload/Harmonic_Drive_Getriebe_DE_1050859_06_2022.pdf.

[5] Newdrive Das Magazin der Harmonic Drive AG. Publisher: LM Druck, Neunkirchen Ausgabe 1/2007. https://cnctar.hobbycnc.hu/VarsanyiPeter/Pdf-ek/HarmonicDrive/ND_0107_df_DE.pdf.

[6] Empower Your Robot. https://www.harmonicdrive.net.

[7] Laifual Drive. https://www.laifualgroup.com.

[8] Bhabani Sankar M., Vineet S., Rathindranath M. (2018). Effect of Cam Insertion on Stresses in Harmonic Drive in Industrial Robotic Joints. Procedia Comput. Sci..

[9] García P.L., Crispel S., Saerens E., Verstraten T., Lefeber D. (2020). Compact gearboxes for modern robotics: S review. Front. Robot. AI.

[10] Kim T., Lee J., Jeong W., Jeon H., Oh S. (2022). Development of Harmonic Drive Combining Four Arcs for Conventional Kinematic Application. Machines.

[11] Wang B., Liu J., Wang C. (2019). Measurement and analysis of backlash on harmonic drive. IOP Conf. Ser. Mater. Sci. Eng..

[12] Pacana J., Markowska O. (2016). The analysis of the kinematic accuracy of the actual harmonic drive on a test bench. Adv. Manuf. Sci. Technol..

[13] Folega P., Siwiec G. (2012). Numerical analysis of selected materials for flexsplines. Arch. Metall. Mater..

[14] Pacana J., Oliwa R. (2019). Use of rapid prototyping technology in complex plastic structures Part II. Impact of operating conditions on functional properties of polymer harmonic drives. Polymers.

[15] Masood S., Song W. (2004). Development of new metal/polymer materials for rapid tooling using fused deposition modeling. Mater. Des..

[16] Oleksy M., Heneczkowski M., Budzik G. (2008). Composites of unsaturated polyester resins applied in Vacuum Casting technology. Polimery.

[17] Pacana J., Oliwa R. (2019). Use of rapid prototyping technology in complex plastic structures. Part I. Bench testing and numerical calculations of deformations in harmonic drive made from ABS copolymer. Polimery.

[18] Pacana J., Pacana A. (2018). Analysis of Possibilities of Using Polymeric Materials for Testing Prototypes of Harmonic Drive. Mater. Res. Proc..

[19] Pacana J., Siwiec D., Pacana A. (2022). New Construction Solutions of Gear Using in Space Vehicle Control Systems. Appl. Sci..

[20] Ren Z., Flašker J. (1996). Numerical stress analysis of harmonic gear drive flexspline. In Proceedings of Seminar Special Mechanicals Gears, Modeling, Product Development and Prospect of Application.

[21] Chuchro M., Czekaj J., Ruszaj A. (2008). Production of functional models and tools by selective laser sintering (SLS, DMLS). Mechanic.

[22] Kalinowski K., Grabowik C., Janik W. (2013). Analysis and testing of the dimensional accuracy of parts made with the FDM technology. Sel. Eng. Probl..

[23] Dudek P. (2013). FDM 3D printing technology in manufacturing composite elements. Arch. Metall. Mater..

[24] Bellini A., Shor L., Guceri S.I. (2005). New developments in fused deposition modeling of ceramics. Rapid Prototyp. J..

[25] Sasimowski E. (2015). Incremental methods of manufacturing elements from polymeric materials. Process. Plast..

[26] Kudasik T., Markowski T., Markowska O., Miechowicz S. (2011). Methods of fabrication of medical models with complex spatial structures. Arch. Foundry Eng..

[27] Rejman E. (2002). Accuracy of models produced in the process of stereolithography. Mech. Rev..

[28] Sobolak M., Rejman E. (2004). Method of Increasing the Accuracy of Stereolitographical Model.

[29] FullCure 720—Material Data, Objet Geometries Ltd. www.2objet.com.

[30] SL5170—Material Data, 3DSystems. www.3dsystems.com.

[31] PA 2200—Material Data, EOS GmbH—Electro Optical Systems PA 2200 Robert-Stirling-Ring 1 AHO/12.08 2/2 D-82152 Krailling/München. https://www.eos.info/03_system-related-assets/material-related-contents/material_pdf.

[32] ABS—Material Data. www.stratasys.com.

[33] Sai Saran O., Prudhvidhar Reddy A., Chaturya L., Pavan Kumar M. (2022). 3D printing of composite materials: A short review. Mater. Today: Proc..

[34] Park S., Shou W., Makatura L., Matusik W., Fu K. (2022). 3D printing of polymer composites: Materials, processes, and applications. Matterials.

[35] Ryan J., Dizon C., Espera A.H., Chen Q., Advincula R.C. (2018). Mechanical characterization of 3D-printed polymers. Addit. Manuf..

[36] Siviour C.R., Jordan J.L. (2016). High Strain Rate Mechanics of Polymers: A Review. J. Dyn. Behav. Mater..

[37] Sobczyk M., Oleksy M., Budzik G., Oliwa R., Stącel M., Majcherczyk H. (2020). Polymers in gearbox production. Polimery.

[38] Johnson M., Gehling R., Head R. Failure of Harmonic Gears During Verification of a Two-Axis Gimbal for the Mars Reconnaissance Orbiter Spacecraft. Proceedings of the 38 Aerospace Mechanisms Symposium.

[39] Ostapski W. (1998). Engineering Design of harmonic Drive Gearings Towards Quality Criteria. Mach. Dyn. Probl..

[40] Mijał M. (1999). Synthesis of Wave Toothed Gears.

[41] Zheng J., Yang W. (2018). Failure Analysis of a Flexspline of Harmonic Gear Drive in STC Industrial Robot: Microstructure and Stress Distribution. IOP Conf. Ser. Mater. Sci. Eng..

[42] Pacana A., Czerwinska K., Bednarova L. (2019). Comprehensive improvement of the surface quality of the diesel engine piston. Metalurgija.

[43] Raviola A., De Martin A., Sorli M.A. (2022). Preliminary experimental study on the effects of wear on the torsional stiffness of strain wave gears. Actuators.

[44] Pacana J., Homik W. (2020). Vibroacoustic testing of prototype hermetic harmonic drive. Sci. J. Marit. Univ. Szczec..

[45] Pacana J., Witkowski W., Mucha J. (2017). FEM analysis of stress distribution in the hermetic harmonic drive flexspline. Strength Mater..

